# The Role of Packaging Size on Contamination Rates during Simulated Presentation to a Sterile Field

**DOI:** 10.1371/journal.pone.0100414

**Published:** 2014-07-08

**Authors:** Tony Trier, Nora Bello, Tamara Reid Bush, Laura Bix

**Affiliations:** 1 School of Packaging, Michigan State University, East Lansing, Michigan, United States of America; 2 Department of Statistics, Kansas State University, Manhattan, Kansas, United States of America; 3 Department of Mechanical Engineering, Michigan State University, East Lansing, Michigan, United States of America; The Ohio State University, United States of America

## Abstract

**Objective:**

The objective of this study was to assess the impact of package size on the contact between medical devices and non-sterile surfaces (i.e. the hands of the practitioner and the outside of the package) during aseptic presentation to a simulated sterile field. Rationale for this objective stems from the decades-long problem of hospital-acquired infections. This work approaches the problem from a unique perspective, namely packaging size.

**Design:**

Randomized complete block design with subsampling.

**Setting:**

Research study conducted at professional conferences for surgical technologists and nursing professionals.

**Participants:**

Ninety-seven healthcare providers, primarily surgical technologists and nurses.

**Methods:**

Participants were gloved and asked to present the contents of six pouches of three different sizes to a simulated sterile field. The exterior of pouches and gloves of participants were coated with a simulated contaminant prior to each opening trial. After presentation to the simulated sterile field, the presence of the contaminant on package contents was recorded as indicative of contact with non-sterile surfaces and analyzed in a binary fashion using a generalized linear mixed model.

**Results:**

Recruited subjects were 26–64 years of age (81 females, 16 males), with 2.5–44 years of professional experience. Results indicated a significant main effect of pouch size on contact rate of package contents (P = 0.0108), whereby larger pouches induced greater rates of contact than smaller pouches (estimates±SEM: 14.7±2.9% vs. 6.0±1.7%, respectively).

**Discussion and Conclusion:**

This study utilized novel methodologies which simulate contamination in aseptic presentation. Results of this work indicate that increased contamination rates are associated with larger pouches when compared to smaller pouches. The results add to a growing body of research which investigate packaging's role in serving as a pathway for product contamination during aseptic presentation. Future work should investigate other packaging design factors (e.g. material, rigidity, and closure systems) and their role in contamination.

## Background and Objectives

Hospital-acquired infections (HAI) have been the cause of substantial pain and suffering of patients, as well as morbidity and mortality [1]. Costs associated with HAIs are not limited to human suffering; they have also had considerable impact on medical expenditures borne by patients, payers and hospitals. Based on 2007 data, Scott [2] suggested that the overall annual direct costs to US *hospitals alone* resulting from HAIs was approximately $28–45 billion.

The US has achieved a significant reduction in HAIs in recent years [3], largely through the focused efforts of the government, researchers, healthcare providers and hospitals. A majority of these efforts have concentrated on reducing direct contact transmission; that is, transmission which occurs between two people, without an intermediate object [4]. Indirect-contact transmissions, those occurring via a contaminated, intermediate object or person, have been investigated by a limited number of studies.

One potential route of indirect contamination involves the contamination of medical devices. Stone et al. [5] reported that a majority of nearly 2 million annual HAIs (based on data collected between1992 and 2000) were associated with the presence of an invasive medical device [6].

Published work focused on medical devices as a potential indirect route for contamination is limited to a study of aseptic technique with surgical clamps [7], threats to sterility from repeated container usage [8], and the evaluation of traffic in the operating room (OR) on contamination of medical devices [9].

Another potential source of indirect contamination is the result of package handling during opening and presentation of devices to the sterile field. Packages that contain sterile devices are only sterile on the inside because the outside has been exposed to non-sterile personnel, environments, etc. As such, any contact of the sterile medical device with the outside of the package, or the hands of the person opening the package, is potentially an indirect route of contamination for the medical device, and, ultimately, the patient.

Sterile items vary significantly by size and shape, are packaged in an array of package types, and these packages can be composed of an array of material. Sterile items are primarily packaged in: (1) flexible pouches made from varying materials or (2) lidded trays (e.g. surgical kits). Materials comprising the sterile barrier system (SBS) may be hydrophilic or hydrophobic; porous or non-porous; transparent or opaque as well as rigid or flexible. Designs may include: peelable or welded seals; have varied seal patterns and composed of single or multiple layers [10]. The contents they contain are equally as variable, encompassing items with differing: profiles, sizes, shapes, components and surfaces, each of which can impact how easily they are packaged and delivered sterilely.

There is a paucity of information on how package factors affect contamination. The few studies that do exist are hampered by small sample sizes and do not directly investigate how differing factors, such as size, influence contamination rates of packaged medical devices. Crick et al. (2008) investigated medical device contamination using a simulated contaminant and five operating room (OR) nurses [11]. The authors performed limited formal analysis, but concluded that the topic should be investigated further. Smith et al. [12] also explored the relationship between contamination, handling and aseptic presentation using surgical screws that were packaged in a double barrier system (non-sterile cover over a sterile package). The authors concluded that the process of opening packaging presents a potential route of contamination. Limitations aside, all studies conducted to date suggest that packaging and the opening process are potential indirect routes for contamination of medical devices.

Herein, we focus our efforts on understanding the effect of packages size on the rate of contact between a simulated device and two non-sterile surfaces (the outside of the package and the hand of the person opening the SBS) using three sizes of flexible peel pouch. Understanding flexible peel packs in the aseptic technique process is relevant to surgical site infections (SSI), but studies of central line catheter kits and removal of the items within may also be relevant to central line associated bloodstream infections (CLABSI). Work has been conducted on the effect of packaging several items together and the likelihood of making technical mistakes [13], but not on the potential for indirect contamination.

**Figure 1:**
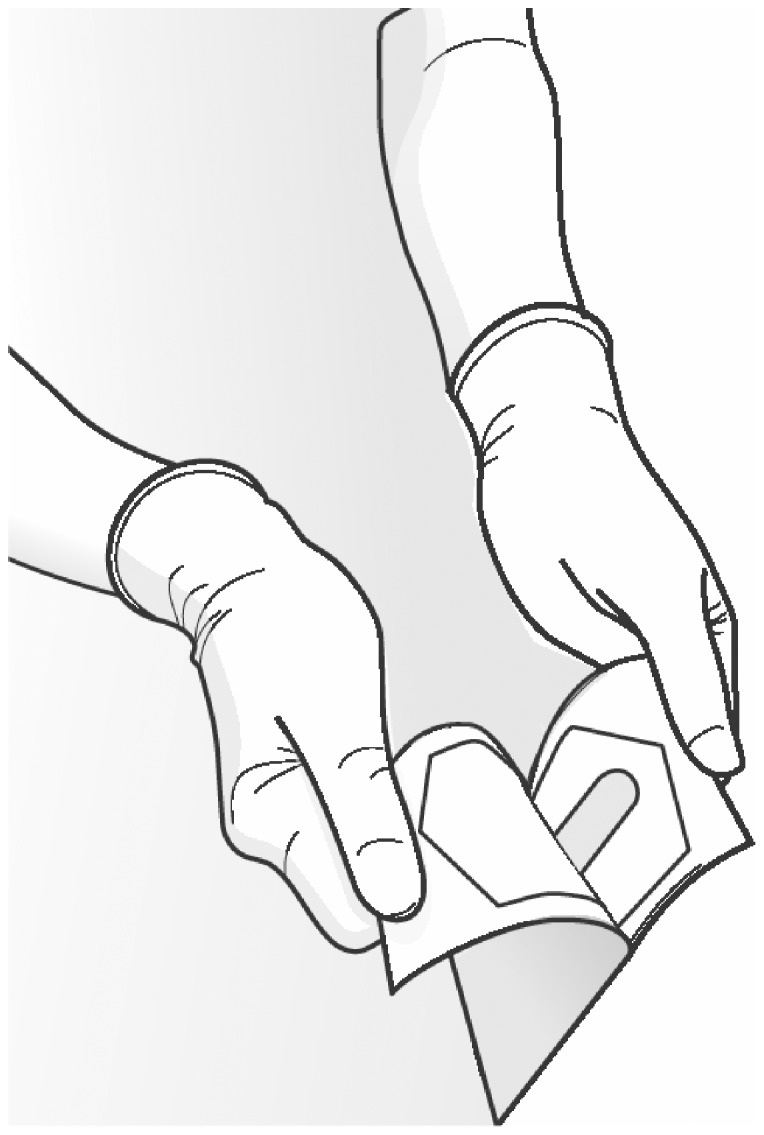
Typical opening approach of a medical device contained in a Chevron Pouch (Adapted from Bix and de la Fuente.

The specific goal of this research was to assess the impact of a single packaging design factor, specifically pouch size in flexible peel packs, on rates of contact between package contents and the outside of the pouch or gloves of the circulator (both of which are considered non-sterile) during aseptic presentation to a simulated sterile field. (See Figure 1).

## Methods

### Ethics Statement

Subjects provided their written informed consent to IRB trained personnel. This study was approved by the Biomedical and Health Institutional Review Board (BIRB) at Michigan State University under approval letter #11–102. All collected data was anonymous.

### Participants

A total of 97 healthcare providers were recruited at three locations, namely the 58^th^ Annual Association of peri-Operative Registered Nurses (AORN) Congress (Philadelphia, PA), the 2011 Annual Meeting of the Association of Surgical Technologists (San Francisco, CA) and the Pontiac Regional Medical Center (Pontiac, MI).

To participate, individuals needed to: be at least 18 years of age; have no history of skin conditions such as latex allergies or eczema; currently be working as, or have worked as, a healthcare professional; and be willing to be digitally video recorded.

Following the informed consent process, participants were asked to complete a survey with demographic information such as age and gender, and to answer several questions regarding their professional experience as a healthcare provider (e.g. title, years employed in healthcare, years of experience with aseptic presentation, etc.). All data were de-identified; information was affiliated only by subject number.

### Pouches

Each participant opened and aseptically presented the contents of three sizes of pouch twice, for a total of six trials per participant. Pouch sizes were: 7.62 cm × 20.32 cm (small), 25.4 cm × 24.45 cm (medium), and 40.64 cm × 26.67 cm (large). To maintain a consistent ratio between the size of the package and the amount of product in the package, pouches were filled with tongue depressors such that the “aspect ratio” (i.e. the pouch area/surface area of the tongue depressors) was equal for all sizes. A target aspect ratio of 6.4 was obtained by including 1, 4 and 7 tongue depressors in small, medium and large pouches, respectively. The length of the medium and large pouches were cut so that their aspect ratios were equal to 6.4, the aspect ratio of the small pouch when it contained one tongue depressor (154.8 cm^2^/24.2 cm^2^).

Tongue depressors were used as the item for presentation because they are inexpensive medical devices with porous properties that contrasted well with the simulated contaminant (see following section). These devices also have a simple physical profile, making it possible to inspect all areas of the device quickly upon completion of each trial. Each pouch was filled with the appropriate number of devices and then sealed using an impulse sealer (Vertrod model 15BP-WE, 126.67°C, 3 seconds).

### Simulation of Contamination

**Figure 2:**
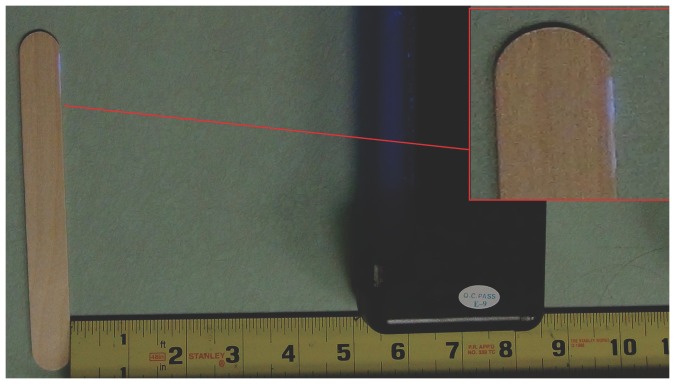
Evidence of Glitterbug presence on tongue depressor as indicator of contact of package contents with the provider's gloves or outside of the package. This photo was taken with a SONY HDR-CX430V video camera using an F-Stop of f/2.4, and an exposure time of 1/30 of a second without flash. The Glitterbug (light blue on the tongue depressor) is illuminated by a black light in ambient lighting.

We adapted the methodology developed by Crick et al. [11] in order to simulate and identify the contact of package contents (i.e. the tongue depressor) with non-sterile objects (i.e. the outside of the package or the hands of the provider). The method utilizes Glitterbug Potion (Brevis Corporation, Salt Lake City, UT) as a simulated contaminant. Glitterbug is commonly used as an educational aid in infection control programs to teach appropriate hand washing procedures. Glitterbug was chosen because it is innocuous, easily transfers to surfaces it contacts and fluoresces upon exposure to UVA black light (Figure 2). Presence of Glitterbug on the tongue depressors was recorded as evidence for contact of package contents with non-sterile objects and used as indicator of contamination of package contents.

### Trials

For each trial, one of the six openings, participants were provided with a new pair of gloves; participants selected their own gloves from sizes that ranged from small to large. The contamination simulant was applied to the exterior of the gloves (with the exception of the pads of the digits involved in opening) immediately preceding a given trial. Test pouches also received a thin coating of the simulated contaminant just prior to being handed to test participants. Coating of the working digits of hand and of the seal area of the pouch were purposefully avoided in order to ameliorate any changes in handling that could have resulted from changes in friction as a result of the simulated contaminant which was a paste-like substance. The gloves and pouch were selected to be coated because these were the two primary contact risks for the package contents (i.e, the tongue depressors).

To minimize the risk of cross contamination, research personnel were assigned to either “clean” or “contaminated” tasks. “Clean” personnel did not handle the simulated contaminant nor any contaminated objects. Their roles were to: prepare drapes (size 72.4 cm × 38.1 cm; Kimberly-Clark, Roswell, GA) representing the sterile field for each trial, record the process with the camcorder, mark the data sheets, and slide the drape carefully into the scanning area. Clean personnel were trained by a single researcher to scan all sides and edges of each tongue depressor, holding the UVA light 3–4 inches away from the itemto assess whether transfer of the simulated contaminant had occurred during presentation. By contrast, “contaminated” personnel were responsible for applying the simulated contaminant, as well as coating pouches and gloves prior to each trial and removing them after each trial.

Each subject participated in a total of 6 trials (two trials with every pouch size), for a total of 582 trials (6 pouches × 97 subjects). Order of pouch size was randomized for each participant to mitigate the effects of fatigue or run order. Immediately following presentation to the sterile field, all tongue depressors were examined under black light for signs of contamination. A trial was recorded as having made contact with a non-sterile surface if at least one of the presented tongue depressors from a package fluoresced upon exposure to black light (example of fluorescence is given is Figure 2).

### Statistical Methods

Data were analyzed to estimate the probability of content contact and to assess the effect of pouch size. For this purpose, the binary response variable (contact: yes/no) was analyzed using a generalized linear mixed model fitted with a logit link function. The linear predictor in this model included the fixed effect of pouch size and the random blocking factor of subject in order to recognize repeated observations on each participant. The cross product of participant and pouch size was evaluated for model inclusion as a random effect but was excluded from the final model as its corresponding variance component converged to zero during the estimation process. Additional explanatory variables, including: gender, education and profession, as well as the covariates age, years of working experience, years of experience with aseptic presentation, hand breadth, and number of repositions, were evaluated for inclusion in the statistical model. Relevance of these explanatory variables to model fit was assessed using maximum-likelihood-based Bayesian Information Criteria and in some cases, P-values. None of these explanatory variables were included in the final model as there was no evidence that any of them contributed to enhance model fit. The final model used for inference was fitted using proc GLIMMIX of SAS (Version 9.2, SAS Institute Inc. NC) with residual Pseudo-likelihood as the estimation method, implemented with Newton-Raphson with ridging as the optimization technique. Results are presented as least square mean estimates (LSME) and corresponding estimated standard errors (SEM). Kenward-Rogers procedure was used to estimate degrees of freedom and to make the corresponding correction in the estimation of standard errors. Post-hoc pair-wise comparisons utilized Tukey-Kramer adjustments to prevent inflation of Type I error due to multiple comparisons.

## Results

### Participant demographics

Data from 81 females and 16 males were available for analysis. Females ranged in age from 26–64 (SD = 9.5 years) and males from 28–62 (SD = 16.2 years). Professional occupation frequencies were: 55 nurses, 40 surgical technologists, one doctor and one “other” healthcare provider. For the purpose of data analysis, the surgical technologists were combined with the two other participants in a “non-nurse” category. Nurses averaged 27.0 years (Range: 3.5 to 44 years, SD 9.1) of professional experience, with an average of 22.8 years of experience presenting to sterile fields (Range: 2.5 yrs to 42 years, SD = 9.9). The participant group comprised of non-nurses reported an average of 14.0 years of experience in healthcare (Range: 0.02 to 44 years, SD = 12.9) and 12.9 years of experience presenting to sterile fields (Range: 0 to 43 years, SD = 12.5).

### Effect of pouch size on content contact

**Figure 3:**
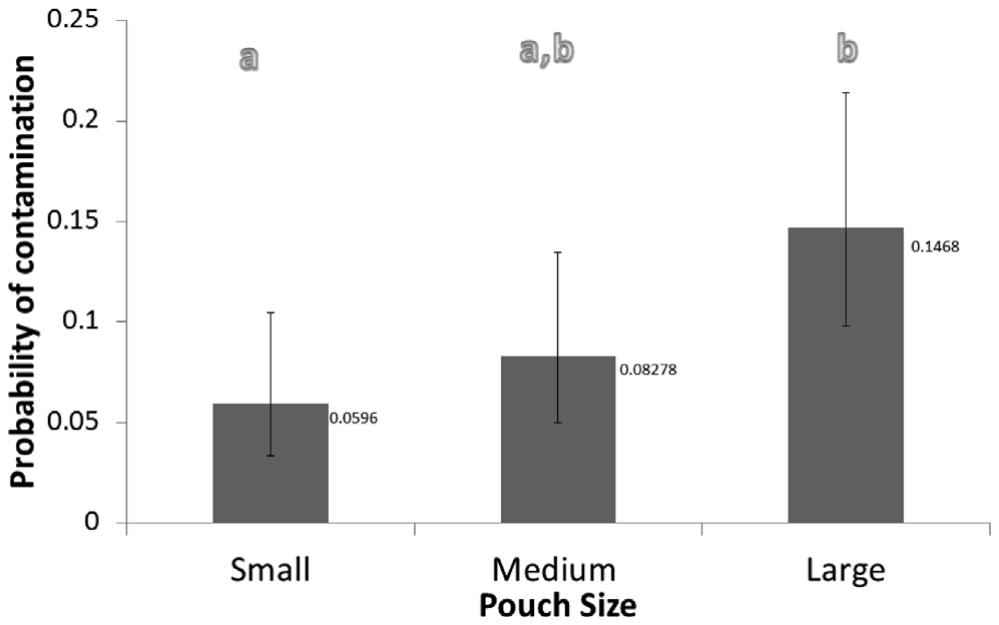
Estimated probability of contact of pouch contents presented to the simulated sterile field from small, medium and large pouches. Whiskers indicate 95% confidence intervals. (a,b) Letters indicate evidence for a significant difference between pouch sizes (P<0.05) (e.g. the small significantly differs from the large but not the medium).

Results indicate evidence for a significant main effect of pouch size on the probability of contact between the package contents and a non-sterile surface during presentation to the simulated sterile field (P = 0.0108). More specifically, larger pouches induced greater rates of contact of package contents than smaller pouches (LSME±SEM: 14.7±2.9% and 6.0±1.7%, respectively; P = 0.0130). Contact rates for medium-sized pouches were intermediate (8.3±2.1%) relative to that of larger and smaller pouches, and there was no evidence of significant difference from either comparison (P = 0.1123 and P = 0.6196, respectively) (See Figure 3).

### Other factors considered

After accounting for pouch size effects, none of the demographic factors considered, namely: gender, age, education, profession, years of working experience, years of experience with aseptic presentation and hand breadth were found to be associated with probability of contamination of package contents during aseptic presentation to the simulated sterile field (P>0.05).

## Discussion

Our study is among the first to examine the impact of package size on successful aseptic presentation of medical devices to the sterile field. Invasive medical devices constitute a potential source of contamination for HAIs [6], as they can serve as a reservoir for opportunistic microorganisms [14]. In particular, the process of package opening during presentation of these devices to the sterile field may be a potential route of contamination [12].

### Testing methodology

Much of the current efforts intended to mitigate the risk of an HAI focus on gloving techniques [15], [16], [17] or effective use of antimicrobials. Given the financial and human burden imposed by HAIs, the study and creation of package designs that facilitate aseptic technique represents an additional area of opportunity in infection control. Our study utilizes the methodology developed by Crick et. al and adapts it. Utilizing the Glitterbug cream is beneficial since it is not visibly apparent that transfer occurred until the UVA light is applied. Over multiple trials, this mitigates the risk of feedback which may bias the results of subsequent trials. This study also utilizes different personnel in specified roles (i.e. clean personnel and contaminated personnel) which help mitigate the risk of cross-contamination and false positives. Herein, we study one aspect of a single design (chevron pouches in three separate sizes), but the method can be applied to quantify the effect that varied aspects of packaging (e.g. design, materials, seal profiles, etc.) have on the potential for contact with contaminated surfaces.

### Effects of package size

Our study provides evidence that pouch size has the potential to impact rates of contamination of package contents during aseptic presentation to the sterile field. Understanding the role of package factors in HAI is a rich area for future investigations. Although the contamination rates between the small and large pouches were significantly different, specific reasons for these differences remain unclear. One theory of the investigative team is that the larger pouches were handled more frequently (i.e. required more repositioning of the hands) during the opening process as compared to the small pouch. In other words, as the participant was opening the small package, he/she would pull it from the center and immediately dump the contents into the simulated sterile field. In contrast, for the larger package, participants often pulled apart one section of the pouch, then slid their hands across the top to pull open another section and repeated this movement until the entire pouch top was open. Through post-hoc analysis of video data, we attempted to characterize differences in handling as a possible explanation. However, the subjective nature of video analysis and of defining what constituted a “hand repositioning event” made quantifying these movements in a repeatable fashion challenging. Further work is currently in progress to better characterize this phenomenon using additional, objective means such as motion capture.

### Limitations

The authors acknowledge that controlling components of the experimental testing conditions, such as the package contents, poses a limitation in the relevance of these results for a realistic surgical setting. For instance, tongue depressors would not usually be packaged in large pouches. However, we chose to use them here to avoid confounding the potential effects of product shape, volume or size with the factor of interest, namely pouch size. Further testing is required to understand the role that the design of the medical device itself may play in rates of contamination (e.g. devices that are thin and flexible like catheters). Additionally, to keep the aspect ratio (surface area of pouch/surface area of product) consistent, the small pouch contained a single tongue depressor, while the medium held four and the large, seven. This resulted in a scenario where the multiple tongue depressors tended to “flow” from the package, while the single one did not. The authors suggest future experiments to determine whether or not the flow of items in this manner impacts contact between product and “non-sterile” surfaces.

Additionally, we limited the scope of the study to the practice of dumping pouch content into the sterile field. Further investigation will need to be conducted to understand how contamination rates for “picking” (transferring a sterile item to sterile personnel who place it in the field) compare to dumping, and the types of packages and devices that should be transferred this way. The authors acknowledge that the simulated contaminate may not be an accurate representation of microbial activity, but that it provides evidence of indirect contact pathways through which such an event could occur. The authors also acknowledge that a positive transfer of the simulated contaminate might not result in an infection; our intention was that it serve as a means to verify contact between the device and a non-sterile surface (i.e. the outside of the package or the gloved hand of the circulator). Finally, the authors acknowledge that the recruitment of subjects attending professional conferences may not be representative of a real scenario in an operating room. However, our setup allowed for assessment of many subjects with high levels of experience. Similar work in a simulated operating room may be worthwhile since operating room traffic and the responsibilities of the healthcare professional may further influence the opening process.

## Conclusion

In summary, this research evaluated the effects of pouch size on contamination rates. The results support the conclusion that larger pouch sizes produced higher contamination rates of the contents. However, the source of the contact (pouch or hands) was not addressed in this work and warrants further investigation.
